# Concurrent bariatric surgery and surgical resection of massive localized lymphedema of the thigh. A case report

**DOI:** 10.1016/j.amsu.2019.10.002

**Published:** 2019-10-11

**Authors:** Loo Guo Hou, Anusha Prabakaran, Reynu Rajan, Fatimah Binti Mohd Nor, Nik Ritza Kosai

**Affiliations:** Department of Surgery, Universiti Kebangsaan Malaysia Medical Centre, Jalan Yaacob Latiff, Bandar Tun Razak, 56000, WP Kuala Lumpur, Malaysia

**Keywords:** Sleeve gastrectomy, Pseudosarcoma, Morbid obesity, Surgical resection, MLL

## Abstract

**Introduction:**

Massive localised lymphedema (MLL) is considered a rare large, pendulous localised benign overgrowth of lymphoproliferative tissue commonly seen in patients with morbid obesity. Histologically, it may be mistaken for well-differentiated liposarcoma; hence, it is also known as pseudosarcoma.

**Presentation of case:**

We describe the successful management of MLL of the left medial thigh in a 35-year-old man weighing 220 kgs (BMI 80.8 kgs/m2). He underwent a concurrent laparoscopic sleeve gastrectomy with surgical resection of the MLL. He recovered well and during our last follow up six months after the operation, he is ambulating well and weighs 148 kgs (BMI 54.4 kgs/m2).

**Discussion:**

MLL is a form of secondary lymphedema resulting in disruption or compression of normal lymphatic drainage due to fat accumulation in obese patients. Patients usually delay treatment for even up to a decade, when it becomes sufficiently large enough to restrict mobility and daily activities, or when it becomes infected. MLL is primarily a clinical diagnosis. A detailed history regarding its slow growth spanning over the years makes malignancy less likely. However, if left untreated, MLL may progress to angiosarcoma. Imaging studies such as computed tomography (CT) and a Magnetic Resonance Imaging (MRI) are usually performed to rule out malignancy or vascular malformations. A tissue biopsy is not recommended unless there are suspicious pigmented lesions.

**Conclusion:**

MLL remains to be underdiagnosed. Due to the obesity epidemic, clinicians must be aware of this once rare disease. The role of concurrent bariatric surgery with surgical resection of MLL warrants further studies.

## Highlights

• Massive localised lymphedema is a rare, benign overgrowth of lymphoproliferative tissue commonly associated with obesity.
• Patients usually delay seeking treatment until it affects their daily activities, or when it becomes infected.
• A tissue biopsy is not recommended unless there are suspicious pigmented lesions.
• MLL remains to be underdiagnosed. Due to the obesity epidemic, it is essential that clinicians be aware of this disease.
• The role of concurrent bariatric surgery with surgical resection of MLL warrants further studies.

## 1 Introduction

Massive localised lymphedema (MLL) is a rare large, pendulous localised benign overgrowth of lymphoproliferative tissue commonly seen in patients with morbid obesity [1]. Histologically, it may be mistaken for well-differentiated liposarcoma; hence, it is also known as pseudosarcoma [2]. Farsid and Weiss first described MLL in 1998 as a lesion caused by chronic lymph obstruction seen in patients with morbid obesity [2]. It is commonly seen in the lower extremities, specifically the medial thighs, but it can develop at other sites including anterior abdominal wall, scrotum, vulva, lower legs, popliteal, groin and penis [3]. MLL located at the retroperitoneal region has also been described [4].

Diagnosis of MLL is primarily clinical, and it presents as a large painless enlarging mass slowly growing over many years [5]. MLL may be misdiagnosed as chronic lymphedema, benign soft tissue mass or even soft tissue sarcoma due to its large size and the rarity of this disease [5]. Keeping this in mind, we report the successful surgical management of MLL of the left medial thigh in a 35-year-old man with a BMI of 80.8 kgs/m2. This case has been reported in line with the SCARE criteria [6].

## 2 Presentation of Case

**Fig. 1:**
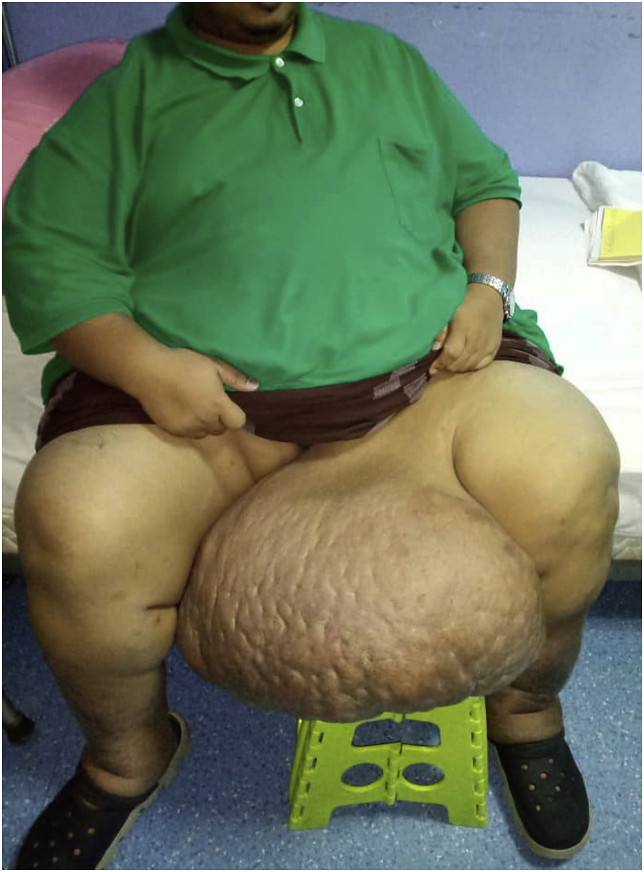
Patient upon clinical examination. A large, pedunculated cutaneous mass is seen at the medial aspect of his left thigh with overlying skin showing peau d’ orange appearance. (For interpretation of the references to colour in this figure legend, the reader is referred to the Web version of this article)

A 35-year-old man with a BMI of 80.8 kgs/m2 (weight of 220 kgs) presented to us with a left thigh mass for the past four years. It has been slowly progressing in size and was associated with recurrent cellulitis requiring multiple courses of antibiotics and dressings. Due to the large size, the soft tissue mass severely restricted his daily activities; hence is he confined mostly to his bed. His medical history includes essential hypertension, ischemic heart disease (New York Heart Association functional class III) and dyslipidemia. On clinical examination, there was a large, pedunculated cutaneous mass located at the medial aspect of his left thigh. It measures 50  cm × 25 cm, and the overlying skin is thickened with peau d’ orange appearance (Fig. 1). We proceeded with ultrasonography of the left thigh mass, which revealed a proximal soft tissue mass over the left thigh with no foci of calcification or cystic component within.

**Fig. 2:**
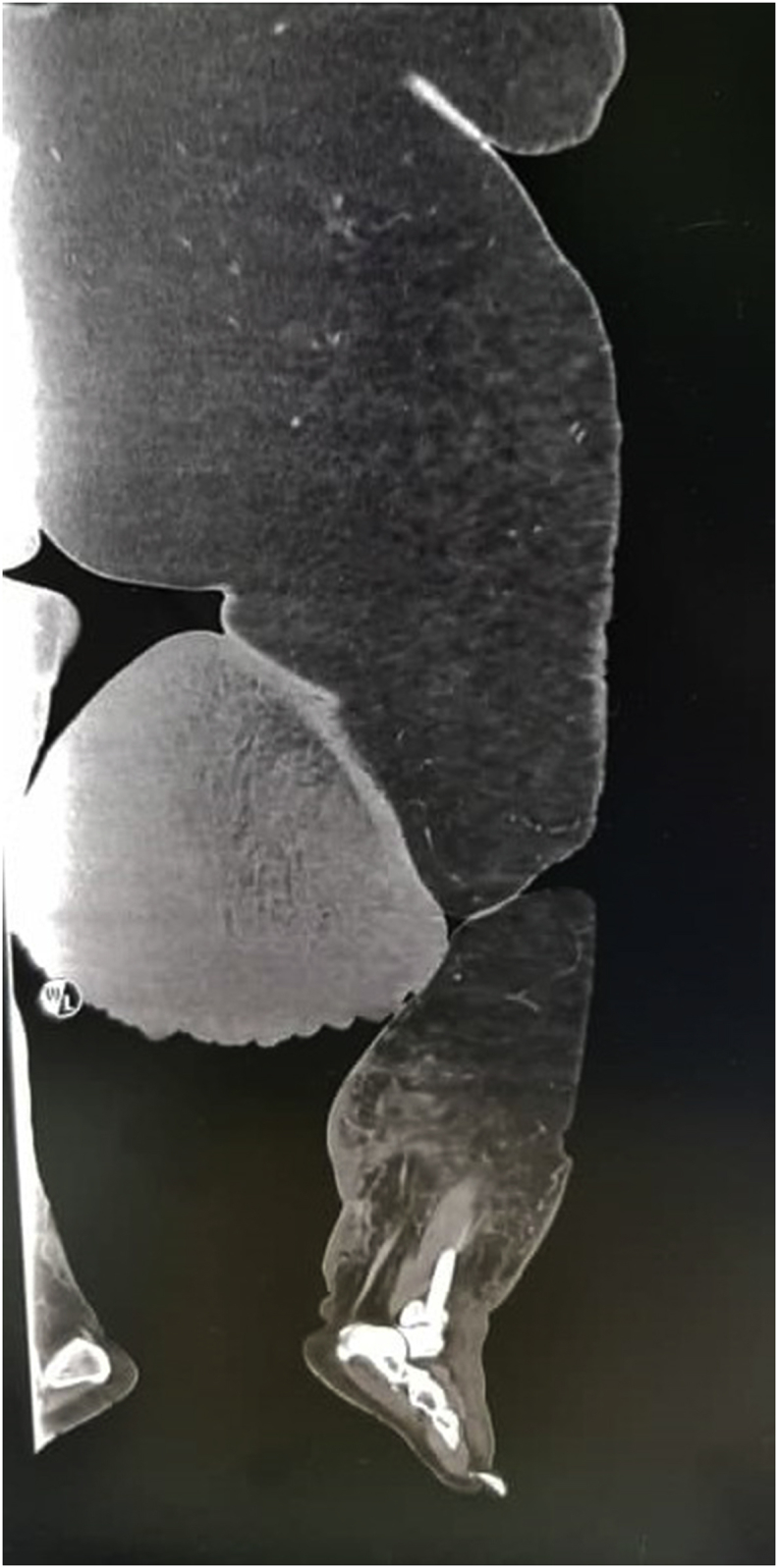
Coronal view of CTA of the left lower limb showing a large hyperdense pedunculated soft tissue mass arising from the medial compartment of the proximal left thigh.

A computed tomography angiography (CTA) of the left lower limb was undertaken to exclude vascular malformation. The CTA showed a large hyperdense pedunculated soft tissue mass arising from the medial compartment of the proximal left thigh. It measures 27x32 × 33cm (AP x W x CC) with collateral arterial supply from the superficial femoral artery (Fig. 2). There were no features to suggest that it was a vascular malformation. After a multidisciplinary meeting, a diagnosis of massive localised lymphedema (MLL) was made. The patient was counselled for a concurrent bariatric surgery with surgical resection of the soft tissue mass.

**Fig. 3:**
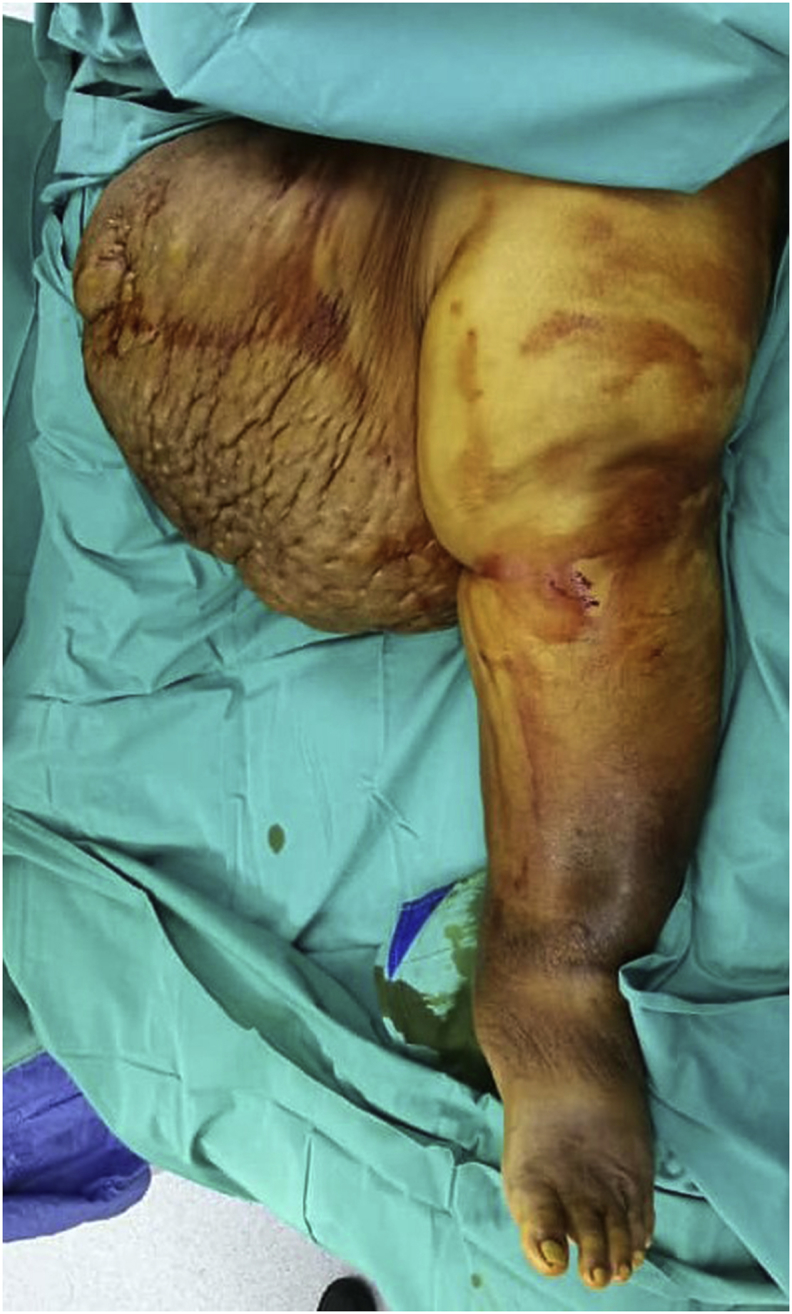
Intraoperative image of the patient on the operating table with the enormous MLL over the left thigh. Resected specimen weighs 16 kgs.

**Fig. 4:**
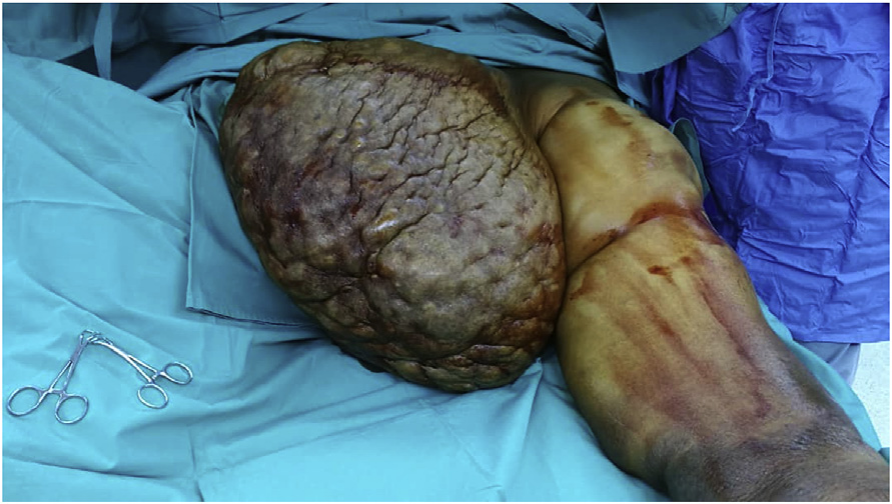
Intraoperative image of the patient on the operating table with the enormous MLL over the left thigh. Resected specimen weighs 16 kgs.

**Fig. 5:**
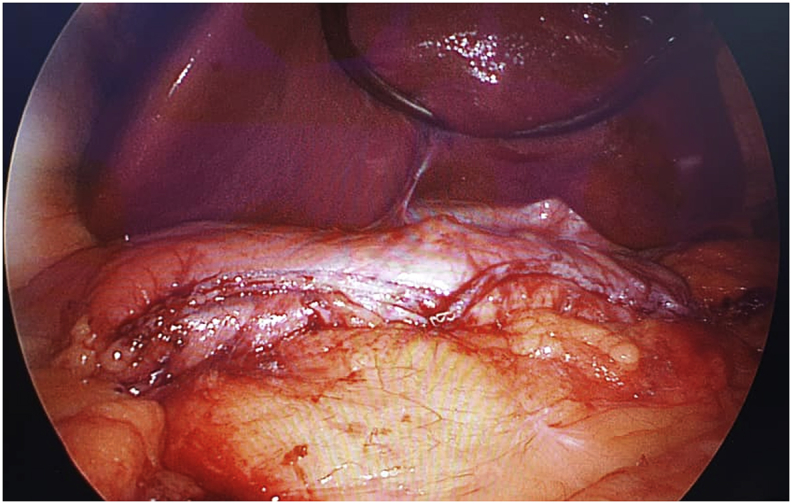
Intraoperative laparoscopic image of remaining stomach after sleeve gastrectomy.

**Fig. 6:**
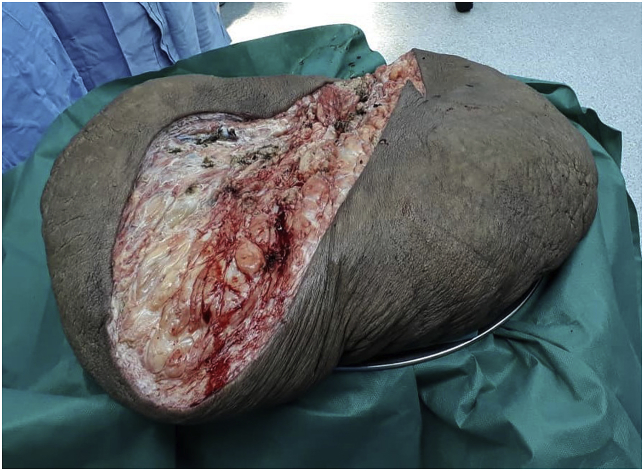
Intraoperative image of resected MLL specimen weighing 16 kgs.

**Fig. 7:**
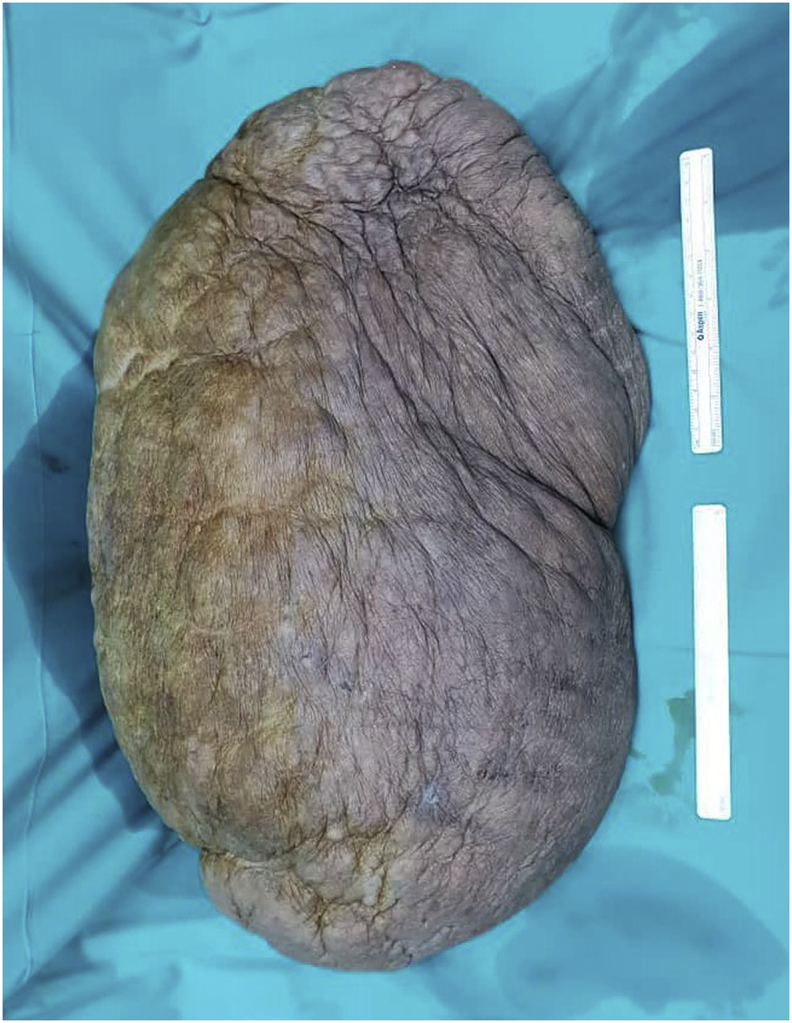
Intraoperative image of resected MLL specimen weighing 16 kgs.

Laparoscopic sleeve gastrectomy (LSG) and surgical resection of the soft tissue mass was performed in the same setting (Fig. 3, Fig. 4). A 5-trocar technique was employed for the LSG. An upper gastrointestinal consultant performed the procedure. A 36 Fr sized bougie was inserted after anaesthetic induction. Standard LSG was performed with a multiple-firing endoscopic stapler device (Fig. 5). A methylene blue leak test was performed. Reinforcement of the stapler line was not performed. A plastic surgeon consultant then performed surgical resection of the MLL. The soft tissue mass weighed 16 kgs and was sent for histopathological examination (Fig. 6, Fig. 7). Postoperatively, the patient was allowed clear fluids on post-operative day one and had fluids only diet for two weeks. He had no abdominal complications, but he developed wound dehiscence over the left thigh wound. The wound subsequently healed with negative pressure wound therapy (NPWT). During his hospital stay, he lost well over 30 kgs, and he was discharged well with physical rehabilitation.

Interestingly, the preliminary histopathology report suggested it to be a well-differentiated liposarcoma. Fortunately, the final report concluded it to be a massive localised lymphedema. During our last follow up six months after the operation, the patient is ambulating well and weighs 130 kgs (BMI 47.8 kgs/m2).

## 3 Discussion

Massive localised lymphedema (MLL) is a rare large, pendulous localised benign overgrowth of lymphoproliferative tissue commonly seen in patients with morbid obesity [1]. It is a form of secondary lymphedema resulting in disruption or compression of normal lymphatic drainage due to fat accumulation in obese patients [3]. Morbid obesity has been shown to cause lower extremity lymphatic dysfunction, which is known as obesity-induced lymphedema (OIL) [7]. MLL therefore is a consequence of OIL, and MLL affects 60% of obese patients with lower extremity dysfunction [7]. It is more common in females, and it has been shown that the average BMI for MLL patients is 60.9 kgs/m2 [3].

The significant and localised lymphedema is so striking in appearance as well as its enormous size that often it is mistaken for a sarcoma [8]. Some authors, however, contend that the pathogenesis of MLL is by a combination of lymphedema and ischemia. The continuous mechanical stretching caused by the weight of the tissue itself is thought to create an ischemic microenvironment, simulating a wound-like process. This, in turn, encourages fibrosis to form and causes lobulation of subcutaneous fat [9].

Patients usually delay treatment for even up to a decade, when it becomes sufficiently large enough to restrict mobility and daily activities, or when it becomes infected [8]. The overlying skin begins to thicken and become keratinised, scaly and cracked after some time, predisposing patients to infection. If left untreated, MLL may progress to angiosarcoma, which is seen in 13% of all reported MLL [5]. Squamous cell carcinoma has also been reported to arise from MLL [10].

MLL is primarily a clinical diagnosis [5,11]. A detailed history regarding its slow growth spanning over the years makes malignancy unlikely. In most cases, a tissue biopsy is not recommended. This is because tissue biopsy is rarely diagnostic, and there is a risk of creating a non-healing ulcer [5,11]. A biopsy may be performed if there is suspicious, pigmented skin lesions [11]. Imaging studies such as computed tomography (CT) and a Magnetic Resonance Imaging (MRI) are usually performed to rule out malignancy or vascular malformations rather than to confirm the diagnosis [5]. However, due to the patient's size and weight, it may be challenging to obtain a CT/MRI scan [11]. We feel that imaging studies may help with surgical planning, as well.

Histopathological findings usually reveal a thickened epidermis, dilated lymphatics, fibrosis in the dermis and extensive interstitial oedema apart from vascular proliferation and inflammatory changes. In deeper soft tissue, fat lobules with thin intervening septa resembling well-differentiated liposarcoma without nuclear atypia may be appreciated [3,8]. One of the distinguishing features is the lack of atypical nuclei, lipoblasts or fibroblasts. This is a salient characteristic distinguishing it from liposarcoma [8]. Often, this lesion has been misdiagnosed as benign soft tissue mass with myxoid changes, benign fibroma or confused for well-differentiated liposarcoma [3]. As in our case, our pathologist initially thought it to be a well-differentiated liposarcoma. Immunohistochemistry study for MDM2 was inconclusive, and after a careful repeat examination, the specimen was concluded to be MLL.

The standard treatment of MLL is complete decongestive physiotherapy (CDP) [11]. This includes pneumatic compression devices (PCD), manual lymphatic drainage, bandaging, skincare and exercise [11]. However, CDP requires experienced therapists to incorporate these massive lymphedemas into bandages. As we do not come across cases like this often, we do not have experienced therapist to perform CDP. Therefore, we elect to perform surgical resection of the MLL alongside a bariatric procedure, which would reduce the risk of recurrence of MLL [11].

A bariatric surgical procedure results in a more significant improvement in obesity-related comorbidities and weight loss when compared to non-surgical interventions [12]. Surgical resection of MLL will allow the patient to be rehabilitated more effectively, prevent recurrent infections, restore mobility and improve patient's quality of life [13,14]. However, complications of surgical resection of MLL such as wound dehiscence, wound infection, and local recurrence has to be taken into account, preferably in a multidisciplinary manner [13].

## 4 Conclusion

MLL remains to be underdiagnosed and underreported in literature. Due to the obesity epidemic worldwide, clinicians must be aware of this once rare entity and have adequate knowledge in its management. MLL should not be mistaken for a malignant neoplasm. As MLL is intrinsically linked to morbid obesity, management of obesity plays a vital role in the prevention of MLL recurrence. Perhaps concurrent bariatric surgery with surgical resection of MLL leads to a better outcome than tackling one at a time conservatively. More studies are required to understand its pathogenesis better, and therefore, the optimal management of MLL.

## Ethical approval

Ethical approval has been exempted by our institution's ethics committee (The National University of Malaysia's Ethics Committee) as this publication is a case report, provided that patients/patient's next-of-kin have given their informed written consent for the publication of this case report.

## Sources of funding

No source of funding.

## Author contribution

Study concepts: Nik Ritza Kosai, Fatimah Binti Mohd Nor, Reynu Rajan, Study design: Nik Ritza Kosai, Fatimah Binti Mohd Nor, Reynu Rajan, Data acquisition: Anusha Prabakaran, Loo Guo Hou, Quality control of data and algorithms: Nik Ritza Kosai, Fatimah Binti Mohd Nor, Reynu Rajan, Data analysis and interpretation: Loo Guo Hou, Anusha Prabakaran, Statistical analysis: -Not applicable, Manuscript preparation: Loo Guo Hou, Anusha Prabakaran, Manuscript editing: Loo Guo Hou, Manuscript review: Nik Ritza Kosai, Fatimah Binti Mohd Nor.

## Registration of research studies

Not applicable.

## Guarantor

Loo Guo Hou.

## Provenance and peer review

Not commissioned, externally peer reviewed

## Consent

Written informed consent was obtained from the patient for publication of this case report and accompanying images. A copy of the written consent is available for review by the Editor-in-Chief of this journal on request.

## Declaration of Competing Interest

None.
